# Sociodemographic characteristics and correlates of Delta-8 THC use in US adults

**DOI:** 10.1192/j.eurpsy.2022.247

**Published:** 2022-09-01

**Authors:** O. Livne

**Affiliations:** Columbia University, Epidemiology, NY, United States of America

**Keywords:** Delta-8 THC, Marijuana, Hemp, Cannabis

## Abstract

**Introduction:**

Recent reports suggest that Delta-8 THC use has surged during the past year. Although Delta-8 THC is believed to have relatively low psychoactive potency, its effects are not well characterized and the associated individual and public health risks are unknown.

**Objectives:**

Identify patterns of Delta-8 THC use among US adult cannabis users and examine associations with sociodemographic characteristics and cannabis use-related variables.

**Methods:**

We surveyed 4,349 US adult cannabis users recruited via online advertisements. We calculated frequencies of sociodemographic characteristics in past 30-day Delta-8 THC users. Odds ratios were used to indicate associations between sociodemographic characteristics and past 30-day Delta-8 THC use.

**Results:**

Respondents aged 45–64 years were significantly more likely than other age groups to have used Delta-8 THC during the past 30 days (odds ratio=1.48, 95% CI 1.04, 2.11). Fifty-eight percent of the sample had heard of Delta-8 THC, 66.8% of which had first heard of it during the last year and 37.1% first heard of it via social media. The most common methods of consumption were vaping concentrates and edibles. We identified motivations for use of Delta-8 THC that potentially indicate a preference for legal options and/or a perception that Delta-8 THC has medical benefits.

**Conclusions:**

This study provides a snapshot of patterns of Delta-8 THC use among US adult cannabis users. Further investigation of the perceived medical benefits of Delta-8 THC, the role of social media in promoting its use, and the effectiveness of restrictions on Delta-8 THC products would provide further information to guide public health policy regarding Delta-8 THC.

**Disclosure:**

No significant relationships.

